# Supplementary material for: Toxicity and Starvation Induce Major Trophic Isotope Variation in Daphnia Individuals: A Diet Switch Experiment Using Eight Phytoplankton Species of Differing Nutritional Quality
Source: Biology (Basel). 2022 Dec 14;11(12):1816. doi: 10.3390/biology11121816 (PMC9775432; doi:10.3390/biology11121816)
Supplement: Supplementary file 1 [file biology-11-01816-s001.zip › biology-2048360-supplementary.pdf]

Supplementary Material

Table S1: Stable isotopic signature of *D. magna* of the different dietary treatments at the last sampling time [h]. Shown are the stable isotopes values, the trophic discrimination factors (Δ_{T4}) at the end of the experiment (96 h) as well as the difference between the end and the initial values (δ_{T4-T0}). For *M. aeruginosa* we used the end values at 72 h (after diet switch) due to high mortality rate of *D. magna* fed on the algae.

Time (h)	diet	$\delta^{13}C$	$\delta^{15}N$	$\Delta^{13}C_{T4}$		$\Delta^{15}N_{T4}$		$\delta^{13}C_{T4-T0}$		$\delta^{15}N_{T4-T0}$	
0	*A. obliquus* (Cyano-Medium)	-26.64 ± 0.19	8.54 ± 0.28								
72	*M. aeruginosa*	*-25.06 ± 0.18*	*9.71 ± 0.72*	7.33 ± 0.29		5.74 ± 0.72		1.57 ± 0.25		1.17 ± 0.59	
96	*P. agardhii*	*-25.90 ± 0.19*	*10.47 ± 0.4*	6.70 ± 0.08	7.15 ± 0.38	6.36 ± 0.40	6.58 ± 1.09	0.74 ± 0.16	0.68 ± 0.82	1.98 ± 0.59	2.42 ± 1.47
96	*P. rubescens*	*-26.91 ± 0.15*	*12.69 ± 1.12*	7.41 ± 0.21		7.64 ± 1.12		-0.27 ± 0.26		5.15 ± 0.85	
96	*T. variabilis*	*-28.78 ± 0.25*	*9.16 ± 0.50*	2.33 ± 0.26		5.58 ± 0.50		-2.14 ± 013		0.61 ± 0.64	
96	*S. elongatus* (green)	-28.14 ± 0.32	9.25 ± 0.57	2.53 ± 0.24	2.17 ± 0.44	5.45 ± 0.57	5.70 ± 0.68	-1.5 ± 0.42	-1.96 ± 0.42	0.79 ± 0.44	0.9 ± 0.82
96	*S. elongatus* (red)	-28.86 ± 0.16	9.92 ± 0.98	1.65 ± 0.03		6.08 ± 0.98		-2.22 ± 0.20		1.37 ± 1.25	
96	*A. obliquus*	*-31.60 ± 0.23*	*8.21 ± 0.10*	1.46 ± 0.16		5.56 ± 0.10		-4.95 ± 0.31		-0.34 ± 0.20	

96	*C. klinobasis*	*-31.45 ± 0.21*	*7.71 ± 0.40*	0.59 ± 0.12	0.84 ± 0.49	5.26 ± 0.40	5.47 ± 0.26	-4.81 ± 0.07	-4.92 ± 0.26	-0.83 ± 0.56	-0.17 ± 0.74
96	*C. vulgaris*	-31.64 ± 0.14	9.19 ± 0.15	0.46 ± 0.2		5.57 ± 0.15		-5.00 ± 0.38		0.65 ± 0.33	
96	starvation	-19.66 ± 0.46	8.06 ± 0.46					1.17 ± 0.59		-0.32 ± 0.51	
